# Transgelin 2 Participates in Lovastatin-Induced Anti-Angiogenic Effects in Endothelial Cells through a Phosphorylated Myosin Light Chain-Related Mechanism

**DOI:** 10.1371/journal.pone.0046510

**Published:** 2012-10-04

**Authors:** Yuan Xiao, Yuhua Li, Jing Han, Yan Pan, Lu Tie, Xuejun Li

**Affiliations:** State Key Laboratory of Natural and Biomimetic Drugs, Department of Pharmacology, School of Basic Medical Sciences and Institute of System Biomedicine, Peking University, Beijing, China; University of Chicago, United States of America

## Abstract

**Background:**

Anti-angiogenic activity is considered to play a key role in the statin-induced anti-tumor effects. We aimed to identify new targets underlying this pleiotropic effect of lovastatin.

**Methodology/Principal Findings:**

We investigated the inhibitory effects of lovastatin on endothelial cell biology and angiogenesis *in vitro*. Lovastatin at high doses inhibited endothelial cell migration and tube formation. Using two-dimensional gel electrophoresis followed by mass spectrometry, we identified the up-regulation of the actin-binding protein transgelin 2 in endothelial cells following treatment with lovastatin. Changes in transgelin 2 levels were confirmed by Western blot and confocal microscopy. We further demonstrated that the Rho signaling inactivation and actin depolymerization contributed to the up-regulation of transgelin 2. The knockdown of transgelin 2 by siRNA dramatically enhanced endothelial migration and tube formation, and meanwhile attenuated the inhibitory effects of lovastatin on cell motility. Moreover, the lovastatin-induced inhibition of myosin light chain phosphorylation was also reversed by transgelin 2 knockdown. The activation of Rho GTPase in the absence of transgelin 2 may represent a mechanism underlying the regulation of phosphorylated myosin light chain by transgelin 2.

**Conclusions/Significance:**

These results strongly imply a novel role for transgelin 2 in the angiostatic activities of lovastatin.

## Introduction

The expansion and propagation of malignant neoplasia are dependent on angiogenesis. Angiogenesis is controlled by a series of steps which involves endothelial proliferation, migration and differentiation. The network of mediators orchestrating this complex process would provide potential targets for pharmacological interventions in tumor therapy [1], [2].

Statins are classical cholesterol lowering drugs. Recent studies have revealed that statins at high-doses possess protective pleiotropic effects against cancer. In addition to the direct effects in killing tumor cells, statins are also reported to inhibit the tumor-induced angiogenesis [3], [4], [5]. Those cholesterol-independent or ‘pleiotropic’ effects of statins are considered to be exerted through the inhibition of Rho family GTP-binding proteins. Given the great variety in the function and distribution of Rho family proteins, it would be important to identify new targets that mediate specific functions underlying those pleotropic effects of statins.

Here we aimed at identifying new targets of lovastatin for its anti-angiogenic activities. Proteomic screening of endothelial cells was performed to identify differentially expressed proteins after lovastatin treatment. The functional significance of the potential targets and the regulatory mechanisms were also investigated. We showed that transgelin 2 was up-regulated by lovastatin, and unveiled a potential pathway for the lovastatin -induced angiostatic effects.

## Results

### Lovastatin inhibits HUVECs migration and tube formation involving the phosphorylated myosin light chain

**Figure 1:**
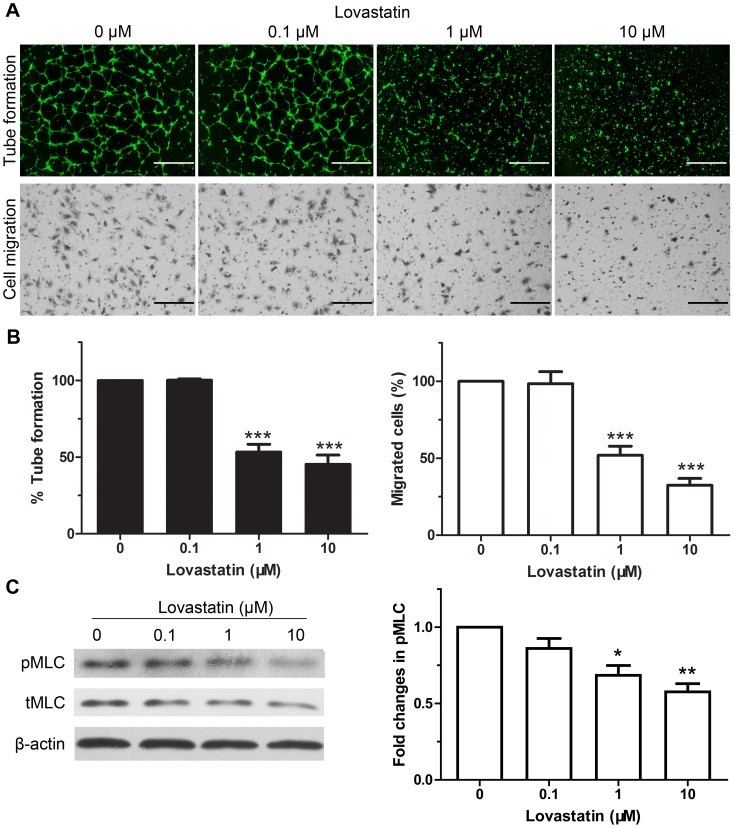
Lovastatin inhibits the angiogenesis and MLC phosphorylation of HUVECs *in vitro*. (A) HUVECs were pre-treated with various concentrations of lovastatin for 24 h. *In vitro* analysis of angiogenesis was carried out using capillary tube formation assay (upper panel; scale bar: 1 mm, ×4 objective) and transwell migration assay (lower panel; scale bar: 200 µm, ×20 objective). Note the dose-related decrease in the relative tube area and migrated cells. (B) Quantitative evaluation of the area covered by tubes (tube formation assay) and relative number of migrated cells (transwell assay) from (A). Data represent four independent experiments. (C) HUVECs were treated as in (A) and analyzed for phosphorylated and total MLC levels. β-actin was also probed as an internal control. Signal intensity of pMLC is expressed as percentage of control intensity ratio. n = 4. Data represent mean ± SEM. *, *p*<0.05, **, *p*<0.01, ***, *p*<0.001 compared with unstimulated control.

*In vitro* studies indicated that statins at micromolar ranges exhibited anti-angiogenic effects [3], [4], [5], [6], [7], [8], [9]. In human umbilical vein endothelial cells (HUVECs), we investigated the effects of lovastatin ranging from 0.1–10 µM. *In vitro* angiogenesis was assessed by Matrigel tube formation assay and transwell migration assay. At 24 h after lovastatin treatment, the tube area in tube formation assays was significantly reduced by lovastatin at 1 and 10 µM, and capillary-like tubes have fragmented and crumbled into cell aggregates (Figure 1). Similarly, in transwell assays, endothelial cells treated with lovastatin at 1 and 10 µM were 48%–68% less motile than control, exhibiting significant motility inhibition.

The myosin-actin machinery powers cell motility. Phosphorylation of myosin light chain is needed for actin-myosin interaction. Figure 1C identified that lovastatin above 1 µM induced significant inhibition of phosphorylated myosin light chain (pMLC) in HUVECs. This is consistent with previous results that statins reduce cell motility partially by pMLC inhibition in a Rho kinase-dependent manner [2], [8], [10], [11], [12].

In clinical trials, the serum levels of lovastatin above micromolar are also readily achievable and generally well tolerated [13], [14]. Therefore, we further investigated the anti-angiogenic effects of lovastatin at the dose of 10 µM, using proteomics approach to characterize novel target proteins.

### Proteomics identification of transgelin 2 as a lovastatin-regulated downstream protein

**Figure 2:**
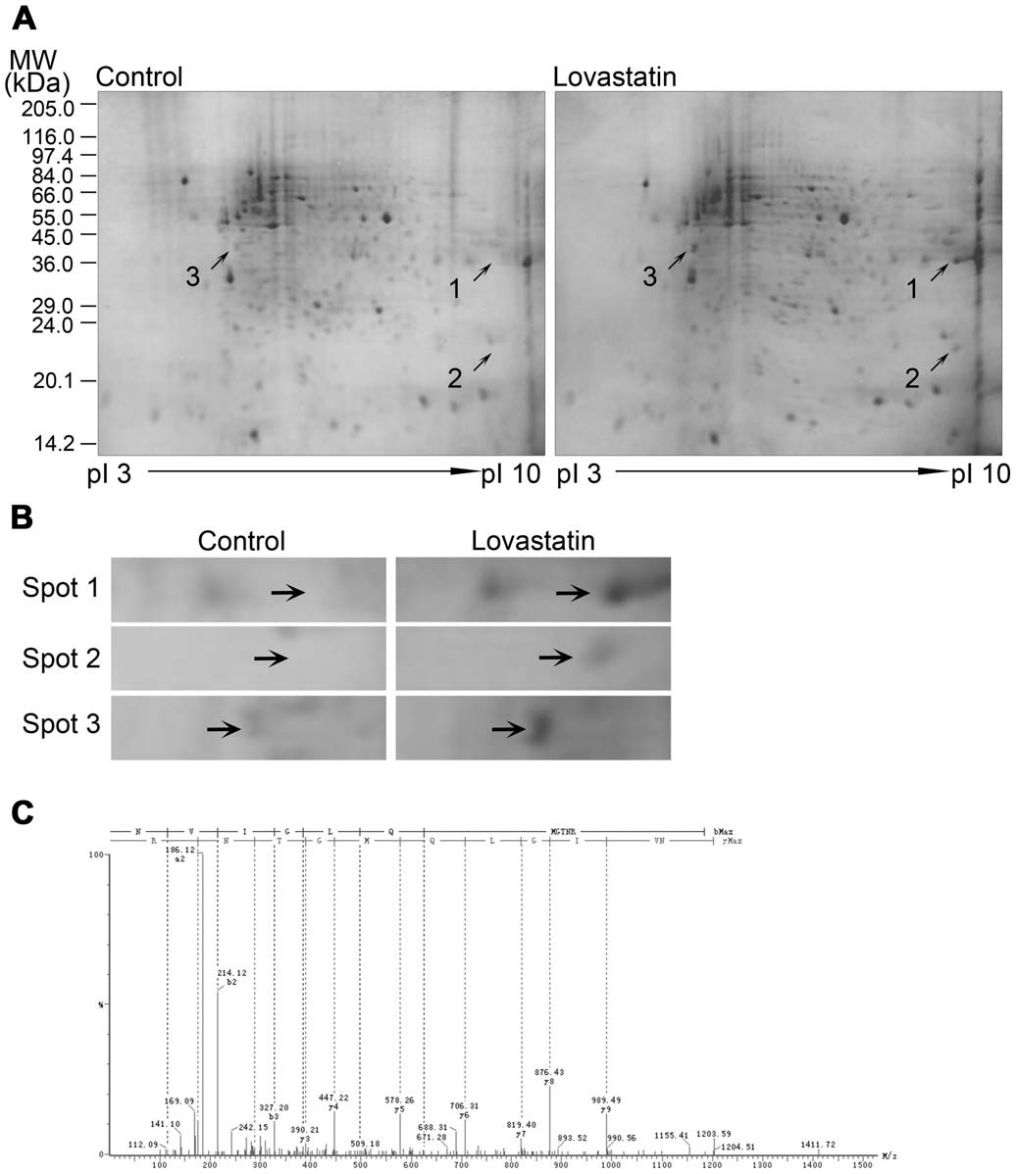
Identification of proteins differentially expressed in response to lovastatin treatment. (A) Representative 2D difference gel electrophoresis (DIGE) images of HUVECs treated with lovastatin (10 µM, 24 h) or vehicle. Among the differentially expressed protein spots, three major spots were selected (arrows). (B) Detailed views of spot 1, 2 and 3 (arrows). (C) The spot 2 was identified as transgelin 2 by ES-Q-TOF-MS analysis followed by database searching, with a Mascot score of 69 and sequence coverage of 16%. The fragment ion spectrum of spot 2 is exhibited.

HUVECs were treated with lovastatin (10 µM for 24 h) or vehicle (DMSO), followed by two-dimensional (2D)-gel separation of cell lysates. Figure 2A shows the representative 2D-gels, about 500 protein spots were detected consistently in each gel by Coomassie blue-staining and 302 spots were matched. Among the differentially expressed proteins, three major protein spots were selected and further analyzed by electrospray quadrupole/time-of-flight mass spectrometry (ES-Q-TOF-MS; Figure 2, B and C). Sequence analysis and database searching identified those proteins as glyceraldehyde-3-phosphate dehydrogenase (spot 1), transgelin 2 (spot 2), and B23 nucleophosmin (spot 3). The up-regulation of transgelin 2 was considered especially noteworthy because of its reported inhibitory role in the malignant phenotype of cancer cells [15], [16], and was thus chosen for further study.

### Immunoblotting identification of transgelin 2 up-regulation induced by lovastatin and through Rho GTPase inhibition and actin disruption

**Figure 3:**
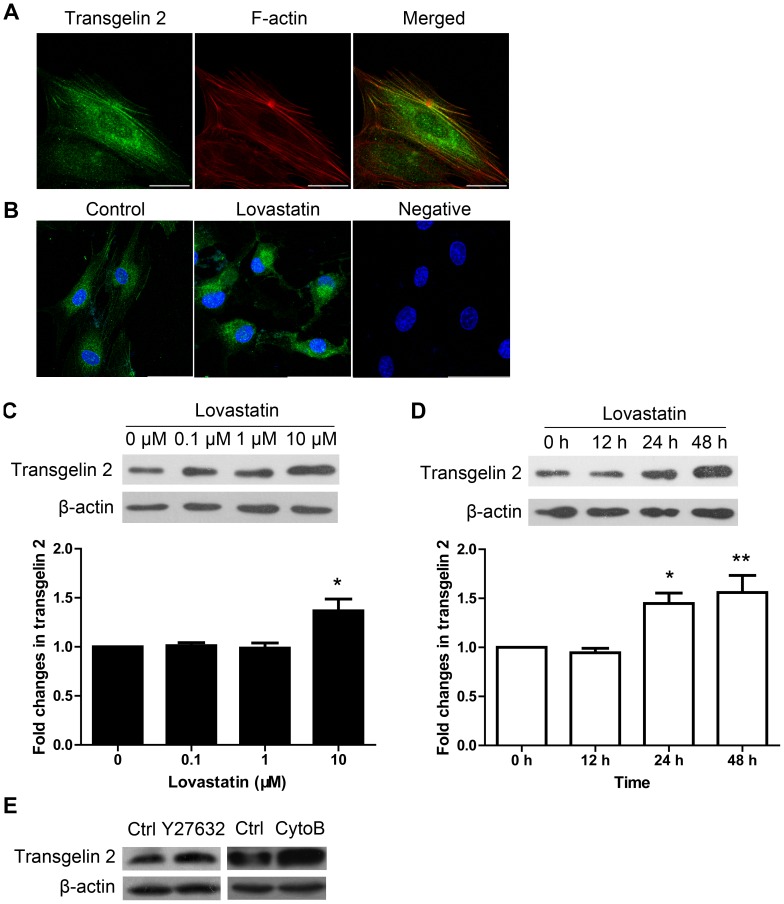
Lovastatin increases transgelin 2 expression. (A) The localization of transgelin 2 in HUVECs. Confocal microscopic examination showed the association (yellow) of transgelin 2 (green) with actin filaments (red). (B) The localization of transgelin 2 (green) in HUVECs after lovastatin treatment (10 µM, 24 h) was visualized under confocal microscope (40× magnification). The negative control shows a confocal image without primary antibodies. Hoechst was used for nuclei staining (blue). Scale bar: 50 µm. (C) Western blot analysis of HUVECs treated with the indicated doses of lovastatin for 24 h. Bands were quantitated by densitometry and normalized against β-actin (n = 3). (D) Western blot analysis of HUVECs treated with lovastatin (10 µM) for the indicated times. Bands were quantitated by densitometry and normalized against β-actin (n = 4). Each data point represents mean ± SEM. *, *p*<0.05; **, *p*<0.01 compared with unstimulated control. (E) Transgelin 2 expression tended to be increased by the ROCK inhibitor Y-27632 (20 µM, 24 h) and the actin polymerization inhibitor cytochalasin B (cytoB, 5 µM, 24 h) compared to control (ctrl).

Transgelin 2 is a homologue of SM22/transgelin and member of a family of actin-binding proteins [17], while its function remains unknown. Confocal microscopic analysis revealed that transgelin 2 was present in the cytoplasm and co-localized with the F-actin cytoskeleton (Figure 3A), which may be indicative for a role in actin-based motility. To verify the up-regulation of transgelin 2 by lovastatin, immunoblotting was used to assess lovastatin's effects. In the dose-dependent study, transgelin 2 expression showed about 37% increase (n = 3, *p*<0.05) over 10 µM lovastatin treatment (Figure 3C). Up-regulation of transgelin 2 expression was also time-dependent (Figure 3D). A significant transgelin 2 increase was observed at 24 h and persisted up to 48 h following lovastatin exposure (10 µM). Meanwhile, we also examined the effect of lovastatin on the cellular localization of transgelin 2 by confocal microscopy. Note the increase in transgelin 2 following 24 h of lovastatin treatment (Figure 3B), which is consistent with the findings from Western blotting.

We next investigated the potential mechanism involved in the regulation of transgelin 2 by lovastatin. Previous studies have indicated the anti-angiogenic effects of statins are mediated by Rho GTPase suppression [7]. To test the effect of Rho kinase inhibition, we treated HUVECs with Y-27632, the Rho/ROCK pathway inhibitor. Y-27632 (20 µM, 24 h) mimicked the effect of lovastatin in up-regulating transgelin 2 expression (Figure 3E). Rho signaling blockade by lovastatin and Y-27632 could induce marked disruption of the actin cytoskeleton [18]. We directly treated HUVECs with cytochalasin B, a well-characterized disrupter of actin filaments. Cytochalasin B (5 µM, 24 h) extensively depolymerized actin filaments in HUVECs (data not shown), and up-regulation of transgelin 2 was detected by Western blot (Figure 3E). The results suggest that the induction of transgelin 2 by lovastatin is mediated through blockade of Rho activation and actin polymerization.

In this study, the dose-response pattern for transgelin 2 expression shifted toward the higher lovastatin concentrations when compared with transwell and tube formation assays (Figure 3 and Figure 1). This is probably due to the implementation of some anti-angiogenic mechanisms that are distinct from actin depolymerization [19]–[20]. We next addressed whether the transgelin 2 regulation does participate in physiological functions that are involved with lovastatin, or it is merely a phenomenon secondary to Rho inhibition. Therefore, we investigated the function of transgelin 2 by RNA interference (RNAi).

### Transgelin 2 participates in angiogenesis and mediates lovastatin-induced migratory inhibition as well as pMLC suppression

**Figure 4:**
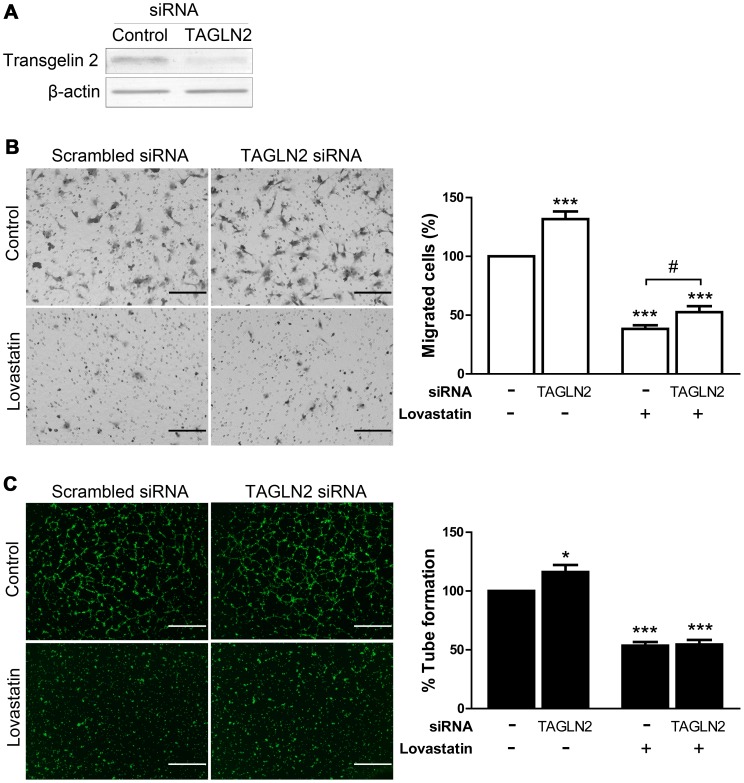
Effects of transgelin 2 knock-down on HUVECs migration and tube formation. (A) Immunoblotting verified the degree of transgelin 2 (TAGLN2) depletion by siRNA. β-actin was employed as loading control. (B) After 24 h of siRNA transfection, cells were treated with lovastatin (10 µM) or vehicle for another 24 h and analyzed for cell migration (scale bar: 200 µm, ×20 objective). Data are evaluated for the average number of migrated cells relative to basal conditions with control siRNA-transfection (n = 6). (C) Cells treated as in (B) were analyzed for tube formation (scale bar: 1 mm, ×4 objective). Data are evaluated for the area covered by tubes relative to basal conditions with control siRNA-transfection (n = 5). Data represent mean ± SEM. *, *p*<0.05; ***, *p*<0.001 versus control siRNA-transfected untreated cells. #, *p*<0.05.

The biological relevance of transgelin 2 in endothelial cells is poorly understood. We conducted transgelin 2 (TAGLN2) RNA interference, and the endothelial functions were analyzed. In the transwell cell migration model, transgelin 2 knock-down markedly increased HUVECs migration by 32% (Figure 4B). Similarly, endothelial tube formation was also increased by transgelin 2 RNAi by 16% (Figure 4C). When combining lovastatin treatment with RNAi transfection, the inhibitory effects of lovastatin in transwell assay was significantly alleviated by TAGLN2 RNAi (Figure 4B). The lovastatin-induced disruption of tube formation was not significantly reversed, apparently due to the overwhelming tube disruption by lovastatin (Figure 4C).

These results demonstrate the function of transgelin 2 underlying lovastatin's effects in angiogenesis. However, no insight into the mechanisms by which transgelin 2 mediates angiogenesis is currently available.

**Figure 5:**
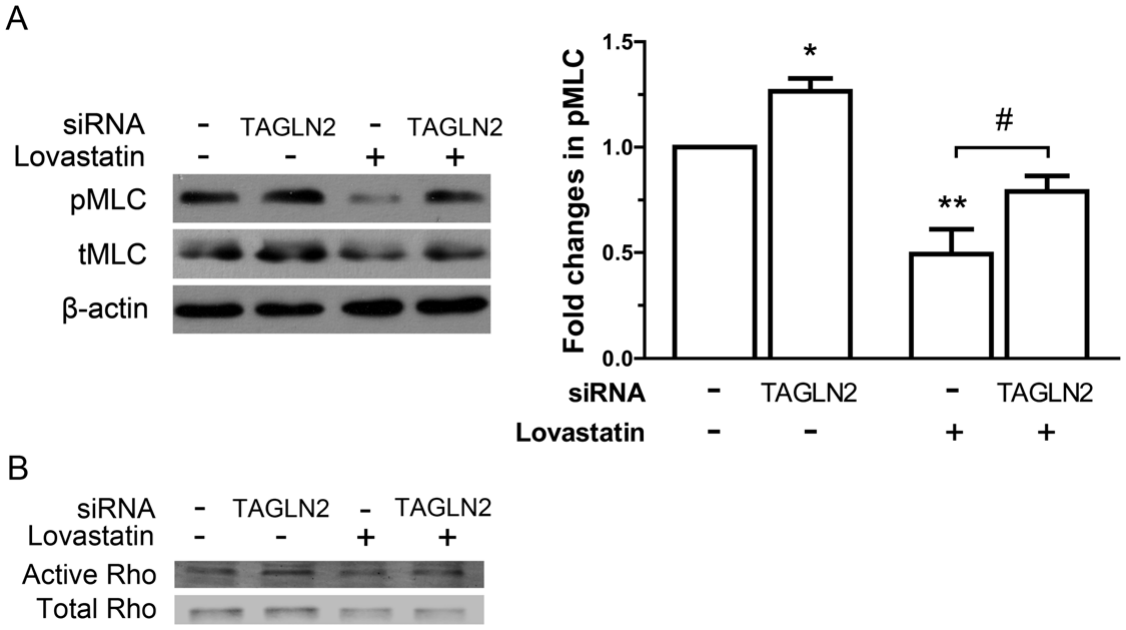
Transgelin 2 regulates HUVECs pMLC levels and Rho GTPases activation. HUVECs were transfected with transgelin 2 siRNA or a scrambled control, and followed by lovastatin (10 µM) treatment as described in Figure 4. (A) Western blot analysis of pMLC and tMLC in HUVECs. β-actin is employed as loading control. pMLC levels were quantitated by densitometry and normalized against β-actin (n = 3). Each data point represents mean ± SEM. *p*<0.05; **, *p*<0.01 versus control siRNA-transfected untreated cells; #, *p*<0.05. (B) Cell lysates were immunoprecipitated with GST-RBD conjugated beads, and active Rho GTPase was detected by immunoblotting. Total cell lysates were subjected to immunoblotting to determine the expression level of total Rho. Experiments were repeated at least three times.

Considering that pMLC is involved in statin-induced angiostatic effects [11], we examined whether it serves as an effector of transgelin 2. We measured the amount of pMLC in TAGLN2 siRNA transfected HUVECs. RNAi knockdown of transgelin 2 led to a 34% increase in the levels of pMLC. Moreover, transgelin 2 knockdown significantly reversed the pMLC inhibition by lovastatin (Figure 5A). The total amount of MLC (tMLC) was also increased following TAGLN2 RNAi.

MLC phosphorylation is under the regulation of Rho GTPase [21], [22]. Indeed, we found significantly increased levels of active Rho GTPase in the absence of transgelin 2, and the lovastatin-induced inhibition was also partially reversed (Figure 5B). This finding was consistent with the changes in pMLC. These data further support the hypothesis that an important function of transgelin 2 is to suppress pMLC activity via Rho signaling, and therefore participating in the pMLC-mediated effects during lovastatin treatment.

## Discussion

The mechanism by which high-dose statins inhibit angiogenesis remains to be fully characterized. In this study, we have demonstrated a role of transgelin 2 in lovastatin-induced anti-angiogenic effects on endothelial cells. To our knowledge, this study is the first to demonstrate a link between lovastatin and transgelin 2, especially for linking transgelin 2 to the lovastatin-induced pMLC inhibition. Furthermore, the identification of transgelin 2 in the regulation of angiogenesis may have important implications for vascular interventions, especially those for cancer treatment.

Transgelin 2 is an actin crosslinking/gelling protein [17], [23], [24]. Using 2D-DIGE proteomic profiling on HUVECs, we identified transgelin 2 as a candidate target of lovastatin. Although only little information about transgelin 2 is available, more is known about its closest homolog, the smooth muscle cell marker transgelin (SM22α), which plays important roles in several cellular events including cell migration and differentiation [23], [25], [26]. The human transgelin 2 protein shares 65% identity and 87% similarity with transgelin in aligned primary sequence space, and has a much wider tissue distribution [17], [27], yet little is known about its cellular functions.

The up-regulation of transgelin 2 was corroborated by Western blot. Meanwhile, studies have reported the down-regulation of transgelin 2 in highly-malignant tumor cells [15], [16], and our study further expands the significance of transgelin 2 down-regulation to the scope of tumor angiogenesis. Further evidences are needed to consolidate the regulation of transgelin 2 in a tumorigenic context, as well as providing an *in vivo* evidence correlating transgelin 2 to the disruption of tumor angiogenesis, and the function of transgelin 2 in microvascular endothelial cells which comprise the tumor vasculature should also be clarified. The cross-system effects of transgelin 2 regulation are also an interesting issue, and comparative analysis between tumor cells versus endothelial cells [28] may provide insights involving the transgelin 2 function in tumor pathogenesis.

In this study, Y-27632 treatment achieved similar effects in transgelin 2 up-regulation as lovastatin. Besides, actin depolymerization by cytochalasin B also caused the transgelin 2 up-regulation. Collectively, these data may imply that lovastatin regulates transgelin 2 expression by acting on cytoskeleton and via Rho inhibition. However, the mechanism underlying these effects leaves many questions open. Either the transcriptional suppression or post-transcriptional regulation is possibly involved, while further evidence is still needed.

Regarding the biological relevance of transgelin 2, endothelial cell motility and tube formation were evaluated in transgelin 2 knock-down cells. The significant increase of endothelial motility is consistent with the recently published findings in Hep3B cells [29], in which study the TAGLN2 knockdown rescued cell motility in PFTK1-supressed cells, and actin polymerization was supposed to be responsible for this effect. However, this observation was in contrast to the study of Yoshino *et al.*, who reported the transgelin 2 RNAi suppressed cell migration and invasion [30]. The current work does not provide a direct explanation for the discrepancy. However, according to unpublished observations, we tend to argue against our results for off-target effects. As regard to neoplastic tissues, the clinical significance of transgelin 2 expression is also controversial. For example, Mareike Elsner *et al.* and Zhang *et al.* have reported opposite results in the relevance of transgelin 2 in tumor malignancy or metastasis [31]–[32]. Therefore additional factors as the tissue- or cell- differences, as well as factors beyond protein expression, may also be concerned in the functional studies of transgelin 2.

The mechanism by which transgelin 2 mediates endothelial angiogenesis remains unclear. In our results, the RNAi study indicates a possible involvement of the phosphorylated MLC. MLC phosphorylation is important for actin-myosin interaction. Homologs of transgelin 2, the calponin family proteins, inhibit actin-myosin interaction without affecting the pMLC levels [33]. Our study provides evidence that transgelin 2, the calponin homolog protein, may directly regulate MLC phosphorylation and total expression. Most noteworthy is the hyper-activation of Rho GTPase by transgelin 2 ablation. Sequence analysis of transgelin 2 identified no recognizable Rho-related domains, therefore more insight should be provided into molecular mechanisms underlying the regulation of Rho activation. The overexpression experiments would also be important to consolidate the putative roles of transgelin 2 in angiogenesis.

Components of the statin-signaling pathways have provided potential targets to inhibit tumor angiogenesis and metastasis. For example, the inhibitors or RNA interference of Rho proteins are designed to improve anti-tumor therapy. However, the complexity of Rho signaling networks has presented challenges for target selection and treatment outcomes [34]. It should also be considered that putative interventions of transgelin 2 might be masked by its homologs. However, interestingly, transgelin 2 has been pointed out to constitute a complementary spatial distribution with its homolog, transgelin, the smooth muscle marker [27]. Zhang *et al.* have also reported the microvessel enrichment of transgelin 2 [32]. Therefore, the expression pattern of transgelin 2 may enlighten its potential for further exploitation.

In summary, this study presents transgelin 2 as a novel target in lovastatin-induced anti-angiogenic pathways. These findings may provide a new avenue for better understanding angiogenesis, and facilitate the development of potential angiostatic therapies especially for cancer.

## Materials and Methods

### Reagents and antibodies

M200 media was from Invitrogen (Carlsband, CA, USA). Lovastatin, Y-27632 and cytochalasin B were from Sigma-Aldrich Co. (St. Louis, MO, USA). Calcein AM and rhodamine conjugated phalloidin were from Biotium (Hayward, CA, USA). Antibodies for transgelin 2 (Proteintech Group, Inc., Chicago, IL, USA), MLCK (Abgent, Inc., San Diego, CA, USA), phospho-myosin light chain 2 and total myosin light chain 2 (Cell Signaling Technology Inc., Danvers, MA, USA) were also purchased from commercial sources.

### Cell culture

Human umbilical vein endothelial cells (HUVECs) were purchased from Invitrogen and maintained in supplemented Medium 200 (Invitrogen/Gibco). All experiments were performed with cells between passages 2 to 5.

### Two-dimensional gel electrophoresis and protein identification

Proteomic analysis was carried out as detailed previously [35]. Monolayer HUVECs were treated with or without 10 µM lovastatin for 24 h and lysed with solubilization buffer. Two-dimensional (2D) gel electrophoresis was performed using immobilized pH gradient strips (pH range: 3–10, Amersham Pharmacea Biotech, USA). Proteins were resolved by isoelectric focusing in the first dimension and SDS-PAGE in the second dimension, and visualized by Coomassie blue R-250 staining. Protein spots were then identified based on their discrete presence in the gel using the PDQuest software (Bio-Rad, Hercules, CA, USA).

The differentially expressed protein spots selected were excised manually from gels and subjected to in-gel digestion. Peptides were extracted from the gel and the protein of interest was analyzed by nanoelectrospray with the electrospray quadrupole/time-of-flight mass spectrometry (ES-Q-TOF-MS, Waters, USA) mass spectrometer. Data resulting from the mass spectrometry analysis were examined with MASCOT search engine (http://www.matrixsciencn.com).

### Cell migration assay

Cell migration was assessed using transwell apparatus (8.0 µm pore size; Corning, New York, NY, USA). The lower compartment was filled with M200 medium containing 10% FBS. The upper compartment was filled with HUVECs re-suspended in M200 medium. After incubation at 37°C for 8 h, cells were fixed with 0.5% crystal violet in 20% ethanol. The migrated cells on the lower surface of the membrane were counted in 10 random fields using a light microscope (×20 objective).

### Matrigel tube formation assay

HUVECs (1.6×10^4^ cells per well) were seeded to the Matrigel (BD Biosciences, New Bedford, MA, USA) coated 96-well plates in Medium 200 (M200) complete growth medium supplemented with 5% FBS. In RNA interference experiments, 1% FBS was alternatively used to reveal more drastic effects of transfection. Cell culture was carried out at 37°C for 4 h. Tubes were stained with calcein AM and observed using an Olympus BX51 fluorescence microscope (Olympus, Tokyo, Japan) with digital camera (×4 objective). Fluorescence images were combined for extended depth of field and processed for analysis.

### Confocal microscopy

Endothelial cells grown on cover slips were fixed in 4% paraformaldehyde and permeabilized with 0.1% Triton X-100 in PBS. After blocking with 5% BSA in PBS for 30 min, incubations with primary antibodies against transgelin 2 were performed overnight at 4°C and conjugated with appropriate DyLight 488-goat anti-rabbit IgG (H+L) secondary antibodies for 2 h at room temperature. To view fibrous actin, cells were stained by rhodamine-phalloidin for 2 h at room temperature. Confocal images were obtained with Leica TCS SP5 laser scanning confocal microscope equipped with a ×40 objective lens.

### Immunoblotting

Cell lysates were collected in RIPA buffer and estimated with the BCA protein assay kit (Pierce, Rockford, IL, USA). Samples were subjected to 12% SDS-PAGE followed by transfer to PVDF membranes. Blotted membranes were incubated with primary antibodies in blocking buffer overnight at 4°C, followed by incubation with secondary antibodies for 1 h at room temperature. The blots were resolved using ECL chemiluminescence kit or alkaline phosphatase detection kit and quantified.

### RNA interference

1.0×10^5^ cells were resuspended with 2 µg of small interfering RNA (siRNA) targeting transgelin 2 (5′-GCAAGAACGUGAUCGGGUU-3′) or a scrambled negative control siRNA in 75 µL of electroporation buffer, followed by electroporation with the Gene Pulser Xcell system (Bio-Rad, Hercules, CA, USA).

### Rho GTPase pull-down assay

Rho activity was assayed by using a Rho activation kit (Thermo Fisher Scientific Inc., IL, USA) according to the manufacturer's protocol. Briefly, cell lysate was incubated with GST-Rhotekin-RBD. Rho-GTP was then precipitated using agarose beads and blotted with anti-Pan-Rho antibodies.

### Statistical analysis

Statistically evaluated data represent mean ± SEM of at least three independent experiments. The statistical significance of differences between groups was calculated by one-way analysis of variance, followed by Newman-Keuls multiple comparison tests to detect specific differences among multiple treatments. Differences of *p*<0.05 were considered significant.
